# PD-1/PD-L1 immune-checkpoint blockade induces immune effector cell modulation in metastatic non-small cell lung cancer patients: A single-cell flow cytometry approach

**DOI:** 10.3389/fonc.2022.911579

**Published:** 2022-09-14

**Authors:** Antonella Fameli, Valerio Nardone, Mojtaba Shekarkar Azgomi, Giovanna Bianco, Claudia Gandolfo, Bianca Maria Oliva, Marika Monoriti, Rita Emilena Saladino, Antonella Falzea, Caterina Romeo, Natale Daniele Calandruccio, Domenico Azzarello, Rocco Giannicola, Luigi Pirtoli, Antonio Giordano, Pierfrancesco Tassone, Pierosandro Tagliaferri, Maria Grazia Cusi, Luciano Mutti, Cirino Botta, Pierpaolo Correale

**Affiliations:** ^1^ Medical Oncology Unit, “Bianchi Melacrino Morelli” Grand Metropolitan Hospital, Reggio Calabria, Italy; ^2^ Section of Radiology and Radiotherapy, Department of Precision Medicine, University of Campania “L. Vanvitelli”, Naples, Italy; ^3^ Department of Health Promotion, Mother and Child Care, Internal Medicine and Medical Specialties (PROMISE), University of Palermo, Palermo, Italy; ^4^ Department of Medical Biotechnologies, University of Siena, Siena, Italy; ^5^ Laboratory of Flow Cytometry, “Bianchi Melacrino Morelli” Grand Metropolitan Hospital, Reggio Calabria, Italy; ^6^ Laboratory of Autoimmunity, “Bianchi Melacrino Morelli” Grand Metropolitan Hospital, Reggio Calabria, Italy; ^7^ Laboratory of HLA Typing and Immuno-Transplantation, “Bianchi Melacrino Morelli” Grand Metropolitan Hospital, Reggio Calabria, Italy; ^8^ Sbarro Institute for Cancer Research and Molecular Medicine and Center of Biotechnology, College of Science and Technology, Temple University, Philadelphia, PA, United States; ^9^ Department of Experimental and Clinical Medicine, Magna Græcia University, Catanzaro, Italy

**Keywords:** immune checkpoint inhibitors, NSCL, flow cytometry, bioinformatics, NKT

## Abstract

Peripheral immune-checkpoint blockade with mAbs to programmed cell death receptor-1 (PD-1) (either nivolumab or pembrolizumab) or PD-Ligand-1 (PD-L1) (atezolizumab, durvalumab, or avelumab) alone or in combination with doublet chemotherapy represents an expanding treatment strategy for metastatic non-small cell lung cancer (mNSCLC) patients. This strategy lays on the capability of these mAbs to rescue tumor-specific cytotoxic T lymphocytes (CTLs) inactivated throughout PD-1 binding to PD-L1/2 in the tumor sites. This inhibitory interactive pathway is a physiological mechanism of prevention against dangerous overreactions and autoimmunity in case of prolonged and/or repeated CTL response to the same antigen peptides. Therefore, we have carried out a retrospective bioinformatics analysis by single-cell flow cytometry to evaluate if PD-1/PD-L1-blocking mAbs modulate the expression of specific peripheral immune cell subsets, potentially correlated with autoimmunity triggering in 28 mNSCLC patients. We recorded a treatment-related decline in CD4^+^ T-cell and B-cell subsets and in the neutrophil-to-lymphocyte ratio coupled with an increase in natural killer T (NKT), CD8^+^PD1^+^ T cells, and eosinophils. Treatment-related increase in autoantibodies [mainly antinuclear antibodies (ANAs) and extractable nuclear antigen (ENA) antibodies] as well as the frequency of immune-related adverse events were associated with the deregulation of specific immune subpopulations (e.g., NKT cells). Correlative biological/clinical studies with deep immune monitoring are badly needed for a better characterization of the effects produced by PD-1/PD-L1 immune-checkpoint blockade.

## Introduction

Non-small cell lung cancer (NSCLC) is the most frequent malignancy worldwide and the leading cause of cancer death (1). In the last few years, mAbs blocking programmed cell death receptor-1 (PD-1) (nivolumab and pembrolizumab) and PD-ligand-1 (PD-L1) (atezolizumab, avelumab, and durvalumab), alone or in combination with chemotherapy, radiotherapy, or bevacizumab, has grown as an established treatment for these patients with remarkable results in term of clinical benefit, progression-free survival (PFS), and overall survival (OS) (2–5). PD-1/PD-L1 immune-checkpoint-blocking mAbs do not exert direct antitumor activity but restore tumor-specific cytotoxic T cell (CTL) function through the inhibition of the immune-suppressive PD-1/PD-L1/2 axis within the tumor microenvironment (6, 7). However, the caveat to this strategy is the risk of unpredictable immune priming with consequent neo-antigen cascade, new clonal T-cell expansion, and functional T-cell immuno-phenotype switch that in turn may lead to worrisome clinical immuno-related adverse events (irAEs) and even death (8, 9).

Therefore, the search for reliable biomarkers to drive the treatment is eagerly encouraged by the scientific community (10, 11). We previously showed that the frequency of mild irAEs and autoantibody [AAb; mainly represented by the extractable nuclear antigen (ENA) antibodies, antinuclear antibodies (ANAs), anti-smooth cell antibodies (ASMAs), and c/p- antineutrophil cytoplasmic antibodies (ANCAs)] increase predict positive outcome in patients with metastatic NSCLC (mNSCLC) and metastatic colorectal cancer receiving immune-checkpoint blockade and chemo-immunotherapy, respectively (9, 12–14). We subsequently, demonstrated that high baseline levels of inflammatory markers (15) and procalcitonin (16) are correlated to a very poor outcome in NSCLC patients on nivolumab or atezolizumab. In the same group of patients, we identified specific class I/II human leucocyte antigen (HLA) alleles involved in CTL-mediated immune reaction and inflammatory response that were statistically predictive of good response to the treatment (17) or predictive of autoimmune pneumonitis (18). In the present study, we have carried out a retrospective immunobiological analysis in a population of mNSCLC patients who have received PD-1/PD-1-blocking mAbs aimed at the identification of specific changes in the peripheral immune-cell population. The changes were subsequently correlated to either an AAb increase or the occurrence of clinically evident irAEs.

## Material and methods

### Patients sample, treatment, and monitoring

This study is part of a retrospective real-world evidence (RWE) multi-institutional study including a total of 119 pretreated mNSCLC patients who had received therapy with nivolumab or atezolizumab, according to the standard international guidelines at the Medical Oncology Unit of The Hospital of Reggio Calabria (OM-RC) and at the Radiotherapy Unit of the University Hospital of Siena (RT-SI), Italy, between September 2015 and April 2020 (16). The current study was focused on 28 of the abovementioned patients who consented to get their blood drawn and anonymously stored for scientific purposes. All procedures were undertaken in compliance with the ethical statements of the Declaration of Helsinki (1964, amended most recently in 2008) of the World Medical Association and respect of their privacy. All patients received PD-1/PD-L1 blockade in a real-world setting as recommended by the international guidelines and regulative agencies following the standard procedures of administration for each drug. All patients had received at least a previous line of platinum-based doublet ± bevacizumab prior to PD-1/PD-L1 blockade. All of them presented with Eastern Cooperative Oncology Group (ECOG) performance status ≤1 and had complete physical examination reports; histological samples; blood cell count; biochemical, immunobiological, and radiological screening; and imaging at the baseline. All patients received the immunological treatment with nivolumab (intravenous infusion of 3 mg/kg every 2 weeks) (16 patients) or atezolizumab (intravenous infusion of 1,200 mg every 3 weeks) (12 patients) until disease progression or occurrence of severe adverse events. Clinical history, physical examination, blood tests, and record of adverse events were evaluated prior to each drug infusion. A computed tomography scan (CT scan) was performed every 3 months or in any case of suspected progressive disease (PD) and evaluated according to the immune Response Evaluation Criteria in Solid Tumors (iRECIST 1.1) (19). All patients were monitored for blood cell count, and biochemistry prior to each treatment course and for their adrenal hormone profile, adrenocorticotropic hormone (ACTH), thyroid-stimulating hormone (TSH), thyroid hormones, anti-thyroid AAbs, ENA antibodies, ANAs, ASMAs, and c/p- ANCAs each month from the beginning of the treatment as reported in previous studies (9, 12).

### Immunophenotyping

Blood was collected into tubes containing ethylenediamine tetra-acetic acid (EDTA) at two time points, baseline and after three treatment cycles. Peripheral blood mononuclear cells (PBMCs) were isolated from blood samples by Ficoll-Hypaque density centrifugation and immediately frozen and stored in nitrogen liquid. Samples were then stained with two different approaches: panel 1 tube, which included markers cluster of differentiation (CD)3 (V450), CD4 (FITC), CD8 [labeled with peridinin chlorophyll protein complex (PerCP)-Cy5.5], CD16 (conjugated with allophycocyanin (APC)-H7], CD19 [conjugated with phycoerythrin (PE)-Cy7], CD56 (conjugated with PE), HLA-Dr (conjugated with PE), and CD45 (conjugated with PE), and panel 2, which included CD3 (conjugated with V450), CD4 [conjugated with fluorescein iso-thiocyanate (FITC)], CD8 (conjugated with PerCP-Cy5.5), CD16 (conjugated with APC-H7), CD19 (conjugated with PE-Cy7), CD56 (conjugated with PE), CCR7 (conjugated with PE-Cy7), FoxP3 (conjugated with FITC), and PD-1 (conjugated with PE), all from BD Biosciences. Samples were acquired on a fluorescence-activated cell sorting (FACS) Canto II flow cytometer (BD Biosciences). This study was not designed to investigate specific myeloid derivative PBMC subsets; however, the presence of monocytes was formally detected in the blood samples by means of forward scatter (FSC), side scatter (SSC), and CD45.

### Data pre-processing and downstream analysis

The data were analyzed with FlowJo (panel 2) or FlowCT (panel 1) (20) (R-based package) for an in-depth study of the cell subpopulation. Specifically, a semi-automated clustering and predictive modeling tool were used for synchronously analyzing 30 FCS files. After pre-processing and quality control of previously FCS files, data were normalized and clustered using self-organized maps (FlowSOM). Cell clusters were represented with uniform manifold approximation and projection (UMAP) dimensionality reduction technique. Cell clusters were annotated based on heatmap clustering, using median values for each FCS. This methodology works better on homogeneous data; thus, we limited this approach to 15 patients from the OM-RC and then manually analyzed the remaining patients from the RT-SI center.

Paired Student’s t-test was used for the analysis of each cluster in the different treatment conditions.

### Human leucocyte antigen genotyping

Samples were processed by the Microbiology Unit Laboratory at the University of Siena and by the laboratory of flow cytometry at the Grand Metropolitan Hospital, Reggio Calabria, Italy. HLA genotyping of the loci A, B, C, and DRB1 was centralized and carried out in the Tissue Typing Unit at the Grand Metropolitan Hospital in Reggio Calabria as previously described by our group (21).

### Statistical analysis

Hazard ratios (HRs) and their 95% confidence intervals were estimated through the Cox regression proportional model; in the multivariate approach, a forward stepwise procedure was used, and the enter and remove limits were set to 0.05 and 0.10, respectively. The association of irAE frequency with biological parameters with clinical outcomes in the two patient cohorts was assessed by the chi-square test. Statistics were performed by the SPSS software 23.0 (International Business Machines Corp., New York, NY, USA) and R statistical software.

## Results

### Study design and patient population

We retrospectively conducted a retrospective bioinformatics analysis on 28 mNSCLC patients (20 men and eight women, four with squamous and 24 with non-squamous histology) who volunteered to provide blood samples for non-profit non-interventional immunobiological analysis. These patients belonged to a larger series of individuals with mNSCLC who received immune-checkpoint blockade at OM-RC and RT-SI according to the standard guidelines for their specific disease. All of them were fit for treatment (ECOG performance status ≤1) and presented no druggable tumor driver mutation/rearrangement (**Table 1**). All 28 patients presented received at least one chemotherapy line with platinum-based doublet ± bevacizumab prior to PD-1 blockade. In this specific series, 16 patients had received salvage therapy with nivolumab or pembrolizumab and 12 with atezolizumab between November 2015 and April 2020 at OM-RC and RT-SI units. During the treatment, after four or five treatment cycles, we recorded one or more irAEs in 14 patients. These adverse events were moderate (G1–G2) in nine patients (skin rash, poly-arthralgia, and thyroiditis) and severe (G3–G4) in further five cases [autoimmune pneumonitis (four cases), myasthenia-like syndrome (one case), hypophysitis (one case), and uveitis (one case)]. The present retrospective study was not empowered to evaluate differences in PFS or OS among patients receiving anti-PD-1 or anti-PD-L1-blocking mAbs. All the patients underwent class I HLA ABC and DRB-1 genotyping and were also monitored for serum levels of inflammatory markers [lactate dehydrogenase (LDH), C-reactive protein (CRP), and erythrocyte sedimentation rate (ESR)], AAbs (ENA, ANAs, and ANCAs) and inflammatory cell counts and neutrophil-to-lymphocyte ratio (NLR) at baseline and after three treatment cycles.

**Table 1:** Patient characteristics at baseline.

Characteristics	Numbers	Percentage
Sex		
***Male*** *** Female***	20 8	71.4% 28.6%
Histology		
***Squamous*** *** Non-squamous***	4 24	14.3% 85.7%
Age		
***<50 years*** *** 51–65 years*** *** >65 years***	3 10 15	10.7% 35.7% 53.6%
Immunotherapy		
***Anti-PD-1*** *** Anti-PD-L1***	16 12	57.1% 42.9%
HLA haplotype		
A*01 or A*02 *** Others*** *** Not determined***	16 5 7	57.1% 17.9% 25.0%
Adverse events		
***G0*** *** G1–G2*** *** G3–G4***	14 9 5	50% 32.1% 17.9%
AAb (ENA/ANA/ANCA)		
***ENA/ANA/ANCA baseline***		
***Negative*** *** Positive***	14 14	50% 50%
***ENA/ANA/ANCA post-treatment***		
***Negative*** *** Positive***	11 17	39.3% 60.7%
Inflammatory markers		
***LDH median value* ± *SD*** ***CRP median value* ± *SD*** ***ESR median value* ± *SD***	341 ± 265 7.9 ± 12.3 41 ± 32.8	

HLA, human leucocyte antigen; AAb, autoantibody; ENA, extractable nuclear antigen; ANA, antinuclear antibody; ANCA, antineutrophil cytoplasmic antibody; LDH, lactate dehydrogenase; CRP, C-reactive protein; ESR, erythrocyte sedimentation rate.

### Effect of therapy on peripheral blood mononuclear cell composition

**Figure 1:**
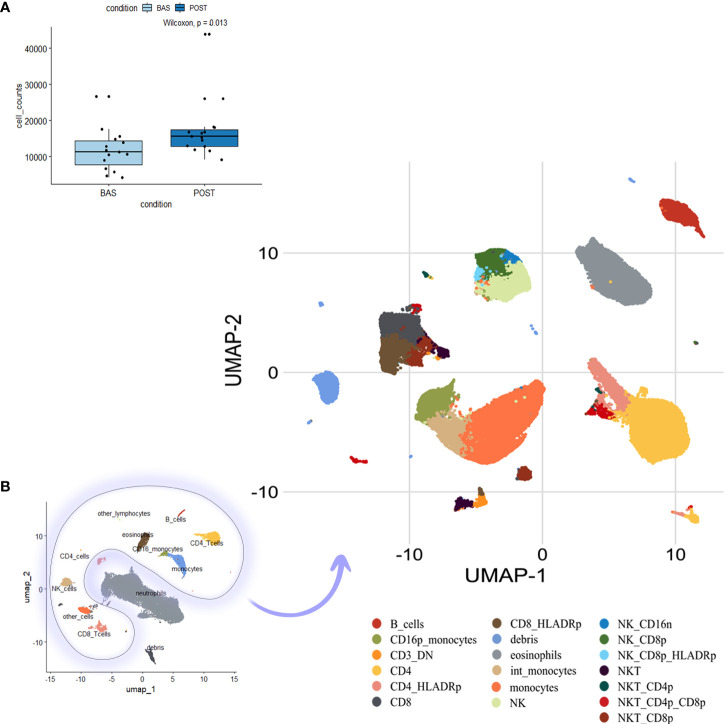
Cell clustering and phenotype identification of PBMCs of mNSCLC patients. Previously compensated flow cytometry standard (FCS) files have been used for large-scale immune monitoring using FlowCT. **(A)** Cell number per time point (basal (BAS) *vs.* post-treatment (POST) after pre-processing, quality control, and normalization. **(B)** Uniform manifold approximation and projection (UMAP) dimensionality reduction techniques for full visualization of 19 independent clusters. PBMC, Peripheral blood mononuclear cells; mNSCLC, metastatic non-small cell lung cancer.

FACS analysis was performed on the PBMCs collected at baseline and after three treatment cycles. We evaluated potential treatment-related changes in different immune populations identified through a multidimensional single-cell bioinformatics approach on 15 patients from the OM-RC center (training dataset). Overall, 435,318 cells were analyzed (**Figure 1A**; interestingly, more cells were recovered after three treatment courses), and, after an automatic clustering approach, we unbiasedly identified 19 sub-clusters of mononuclear cells based on markers profile (**Figure 1B**), namely, B cells, CD16p^+^ monocytes (non-classical monocytes), CD3^+^DN (double negative), CD4^+^, CD4^+^HLADR^+^, CD8^+^, CD8^+^HLADr^+^, Debris, Eosinophils, intermediate (int) monocytes, monocytes (classical), NK, NK_CD16^−^, NK_CD8^+^, NK_CD8^+^HLADR^+^, NKT, NKT_CD4^+^, NKT_CD4^+^CD8^+^, and NKT_CD8^+^ (**Supplementary Figure 1** represents a heatmap reporting the expression of each marker in each identified cell population, and most populations have been identified thanks to their unique combination of marker expression, e.g., neutrophils were FSC/SSC high, CD45mid, CD16dim, and negative for all other markers).

**Figure 2:**
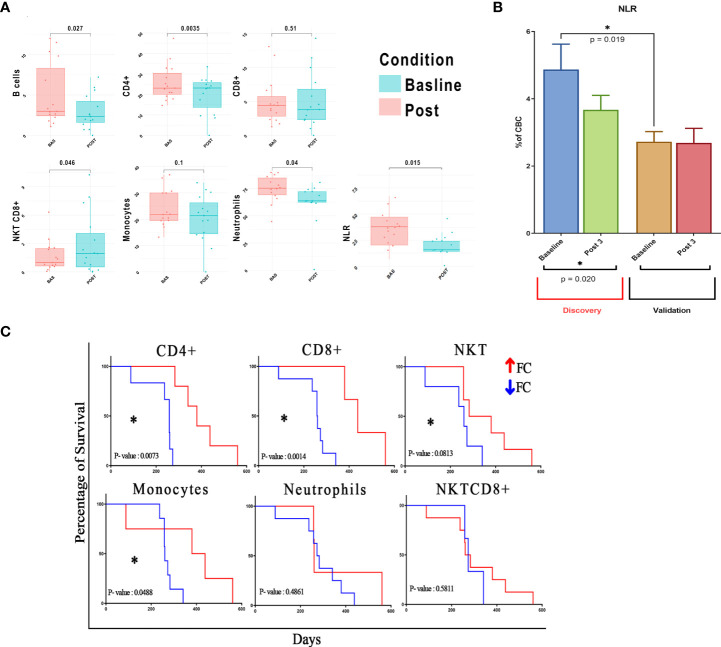
Correlation of different cell clustering and mNSCLC patient survival. Post-treatment fold change of previously identified cells is calculated and used for downstream analyses. **(A)** Values of seven fundamental cell clusters before and after therapy are shown as grouped dot plot, paired Student’s t-test is used for the analysis of each cluster in the different treatment conditions, and clusters with p-value < 0.05 were considered as significant. y-Axis represents % of cells on total lymphocytes for B/T/NKT cells, total cellularity for monocytes and neutrophils, and an absolute number for NLR. **(B)** NLR calculation in two different study centers to demonstrate baseline differences. **(C)** Survival analysis based on population fold changes; p-value < 0.1 considered as trend, and p-value < 0.05 considered as statistically significant. mNSCLC, metastatic non-small cell lung cancer; NLR, neutrophil-to-lymphocyte ratio. *= pvalue<0.05.

After three treatment courses, we observed a significant decline in CD4^+^ T cells, B cells, and neutrophils [absolute mean difference (MD) = −4.46%, p-value = 0.0035; MD = −2.5%, p-value = 0.027; and MD = −13.03%, p-value = 0.04, respectively) and a decreasing trend for monocytes (MD = −3.88%, p-value = 0.1). Additionally, we recorded an increase in NKT_CD8^+^ cells (MD = 1.31%, p-value = 0.046) (**Figure 2A** and **Supplementary Figure 2**). Likely due to the small sample size, only CD4^+^ T-cell decrease was validated in the dataset of patients from the RT-SI center (**Supplementary Figure 3**). As shown in **Figure 1B**, all the cell populations were additionally clustered separately from neutrophils. Interestingly, a significant decrease in NLR (calculated by FACS analysis) was observed after three treatment cycles with a p-value = 0.015 (**Figure 2A**), perfectly overlapping the changes in the peripheral blood cell count analysis (**Figure 2B**). Again, this aspect was not validated in patients from the RT-SI center (**Supplementary Figure 3**).

Additionally, we found a significant difference in the baseline values of NLR between patients from the two centers (mean 4.87 vs. 2.72 for OM-RC *vs.* RT-SI, p = 0.019) (**Figure 2B**). Interestingly, this difference disappeared after three treatment courses of anti-PD1/PD-L1 mAbs, thus ruling out the hypothesis of a laboratory-related batch effector or other possible confounding factors (sex, age, previous radiation therapy, and histology). Another plausible hypothesis may be formulated on the fact that OM-RC and RT-SI centers adopted different frontline treatments prior to immunotherapy. In fact, all of the patients enrolled in RT-SI had received frontline chemotherapy with fractioned cisplatin and oral metronomic etoposide ± bevacizumab (mPE/mPEBev), a very active regimen with powerful immunomodulating activity (22–26). On the contrary, the majority of patients enrolled at OM-RC had received more conventional frontline chemotherapy doublets (either carboplatin and taxol or carboplatin and pemetrexed). Therefore, all of the patients in the validation group derived from RT-SI had received a frontline systemic treatment with the potential ability to impair the myeloid and inflammatory components due to the long-term sequestering activity of vascular endothelial growth factor (VEGF) exerted by bevacizumab together with the immunobiological effects of metronomic chemotherapy as reported in previous studies (22, 23, 27). Considering the prognostic value of blood cell subsets in patients with NSCLC, we attempted to build a risk model to deeply evaluate their impact on NSCLC patient outcomes. Thus we conducted a statistical analysis based on fold-change modifications after three treatment cycles. Our multidimensional analysis of PBMCs showed no significant post-treatment changes in CD8^+^ T cell abundance but significant changes in CD4^+^ and B cells (p < 0.05) according to Student’s t-test (**Supplementary Figure 3**). Interestingly, we found a significant correlation between pretreatment atypical monocytes (negative correlation), CD3^+^CD4^−^CD8^−^ (positive correlation), and CD8^+^HLADR^+^ (positive correlation) NKT cells (positive correlation) and survival (**Supplementary Figure 4** and **Supplementary Table 1**). In line with what was previously observed, we found that patients with significant post-treatment increases of CD4^+^, CD8^+^, and monocytes showed a significant improvement in survival according to the log-rank (Mantel–Cox) test (p < 0.05). Similarly, an increase in NKT also showed a clear-cut trend to improve the outcome (**Figure 2C** and **Supplementary Figure 5**) of these patients. None of the other cell subset treatment-related changes was correlated with patient survival (**Supplementary Figure 5** and **Supplementary Table 1**).

**Figure 3:**
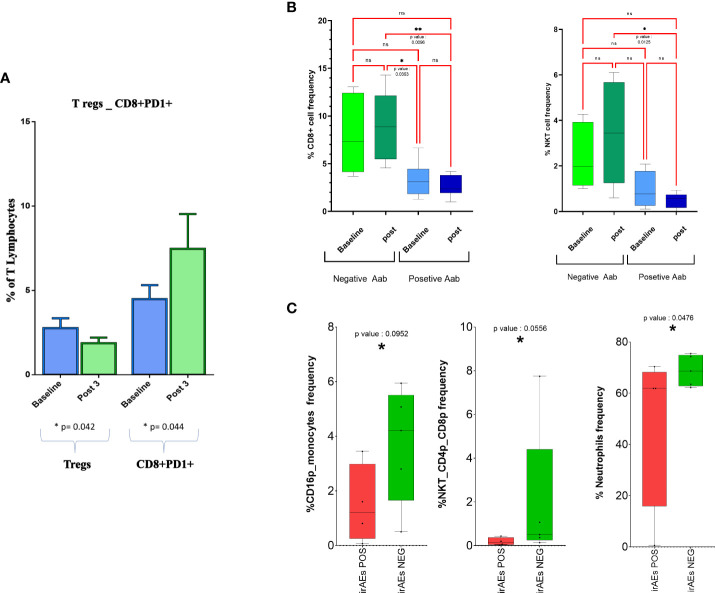
CD8^+^ T cells and NKT cell frequency changes in response to treatment in groups with different inflammatory and autoimmunity markers. **(A)** Boxplot representing changes on peripheral blood T_reg_s and PD-1^+^CD8^+^ T lymphocytes as effect of treatment. **(B)** Total percentage of different cell subsets before and after therapy is reported for patients positive and negative for antinuclear antibodies (ANA, AAbs); only significant results are indicated. **(C)** Abundance of different cell subsets before and after therapy is reported for patients experiencing or not irAEs. Cell subsets with p-value < 0.1 only are represented. NKT, natural killer T; irAEs, immuno-related adverse events. *= pvalue<0.05. , **= p value<0.01 ns, not significant.

For a small group of 12 patients who received treatment at RT-SI, we had sufficient material to evaluate possible changes in peripheral central memory (T_CM_; CD3^+^CD8^+^CD45RO^+^CCR7^+^) and effector memory (T_EM_, CD3^+^CD8^+^CD45RO^+^CCR7^−^) T cells, T_reg_s (CD4^+^/CD25^+^/Foxp3^+^ T cells), and PD1^+^ CTLs. In this very small cohort of patients, we recorded no change in T_CM_ and T_EM_ subsets, a significant decline in T_reg_s, and a paradoxical increase in CD8^+^PD1^+^ T lymphocytes after three treatment courses of nivolumab (**Figure 3A**).

### Inflammatory profile, autoimmunity markers, and immuno-related adverse events

In the present set of mNSCLC patients, we recorded a significant treatment-related appearance and/or increase of serum ANA (baseline *vs.* the third cycle = 28% *vs.* 64%, p < 0.0005), while no statistically changes were observed in terms of inflammatory markers (CRP, ESR, or LDH) (p > 0.5). This finding was in line with what was reported in previous studies, sustaining the good quality of the results (9). Overall, in our small set of 28 patients, there was no change in white blood cell counts with the exception of eosinophil cell counts, which were significantly increased in the post-treatment setting [140 ± 130 *vs.* 240 ± 180 cells/µl; p = 0.0022]. Also, these results were once again consistent with what was shown in previous studies (24).

Our analysis revealed a much lower frequency of CD8^+^ and NKT cells in patients who were positive for ANA screening (**Figure 3B**) compared with those who remained negative for the expression of these AAbs along the immunological treatment. On the contrary, we were unable to find any other immunological difference between the two cohorts of patients (**Supplementary Figure 6**).

We also evaluated the occurrence of possible changes in specific immune populations in patients who had or did not have irAEs in the course of the treatment. Interestingly, we found an increase in neutrophils, CD16^+^ monocytes, and NKTCD4^+^CD8^+^ cells only in those patients who presented irAE signs (p < 0.1) (**Figure 3C** and **Supplementary Figure 7**).

Finally, we were unable to find any correlation neither between survival and specific HLA haplotypes nor with HLA and specific immune cell changes (**Supplementary Figures 8** and **9**) due to the small available dataset that was unable to fulfill the statistical analysis requirements.

## Discussion

We previously showed that PD-1/PD-L1 immune-checkpoint blockade with nivolumab or atezolizumab is often associated with an increase in AAbs (ENA, ANAs, and ASMAs) and clinical irAEs in mNSCLC patients (9). We showed that these events are predictive of positive outcomes and prolonged survival (9). Our findings were in line with the literature regarding the general use of immunotherapy and immune-checkpoint inhibitors in cancer patients (9, 12, 24). In the present series of 28 patients who volunteered for deeper immunobiological monitoring, we found that the treatment with anti-PD-1 (nivolumab) or anti-PD-L1 (atezolizumab) mAbs produced similar effects in the peripheral blood and was similarly associated with a significant decrease in neutrophils, CD4^+^, and B cells coupled with an increase in NKT-CD8^+^ cells and eosinophil cell counts. Further T-cell subset analysis showed a significant decline in immune-suppressive T_reg_s in the 12 patients who received nivolumab at the RT-SI center. In the latter patients, we also recorded a treatment-related increase in the percentage and the absolute number of peripheral CD8^+^PD-1^+^ T cells [baseline *vs.* post-treatment: 4.40% *vs.* 6.02%, p = 0.04). Also, the latter finding was perfectly in line with what was reported by other authors (25). We believe that the increase in PD-1^+^ T cells could be a potential mechanism of resistance to PD-1 blockers in the long term and should be investigated in more extensive perspective studies. However, in the present investigation, we also found that treatment with PD-1/PD-L1 immune-checkpoint inhibitors significantly reduces the NLR in these patients. This finding suggests the additional ability of this new class of drugs in modulating the systemic inflammatory asset as reported in other studies (16). It has been in fact hypothesized that PD-1/PD-L1 blockade induces enforcement in the effector CTL compartment, which is counteracted by a consequential decline in neutrophil and T_reg_ counts. The latter event might theoretically be considered a natural negative feedback response to an enhanced activity of CTLs rescued by the PD-1/PD-L1 blockade (26, 28). It is noteworthy that our results provide the first evidence in NSCLC that a previous antiangiogenic/immunomodulatory treatment (bevacizumab and metronomic therapy in our case) could significantly affect the response to the next immunotherapy. This is consistent with our previous data and hypotheses and once again evidences a very strong VEGF and immune-checkpoint axis, which warrants further investigation (22, 23, 27).

Furthermore, the effect of PD-1 blockade on the innate immunity of our patients unraveled a significant treatment-related impact on the NKT compartment. NKT cells are cells at the “bridge” between innate and adaptive immune response, whose activation depends upon the engagement of their T-cell receptor (TCR) by the major histocompatibility complex (MHC)-like CD1d loaded with lipids, given that, once activated, these cells could produce various cytokines, such as interferon (IFN)γ, interleukin (IL)4, IL2, IL10, and IL21, and reprogram dendritic cells to produce IL12. It is conceivable that (as reported in multiple preclinical and clinical models of cancer immunotherapy, including melanoma) the treatment with immune-checkpoint inhibitors could activate NKT cells, which in turn rescue tumor-specific CTLs from exhaustion (29, 30). These data are consistent with our results showing that NKT increase was associated with a trend to improved survival, further supporting the idea of a broader microenvironmental immune re-modeling impacting patient outcomes. Finally, we investigated the possible immunobiological changes occurring in the presence of treatment-related autoimmunity/irAEs in these patients. We confirmed the lack of a specific treatment-related peripheral blood immune profile able to predict the occurrence of either AAb increase or irAEs. Nevertheless, we found that the risk of irAEs in the course of the immunological treatment is associated with a reduced percentage of myeloid and NKT subpopulation (atypical monocytes and double CD4CD8-positive NKT) in the peripheral blood. The results of other authors also suggested a positive correlation among CD4CD8-double negative T cells, CD8^+^HLADR^+^ T cells, and NKT-like cells and survival, thus proposing that cell lineages have a role as potential biomarkers. In particular, a similar pattern associated with thyroid gland infiltration has been described in patients developing thyroiditis in the course of the treatment with PD-1 or PD-L1 inhibitors. This finding pictures possible immunobiological changes associated with treatment-related autoimmunity/irAEs occurring in patients receiving immune-checkpoint-blocking mAbs and also hypothesizes a potential link between the occurrence of irAEs (specifically thyroid gland-specific autoimmunity) and improved outcome (31).

Overall, the findings from this proof-of-concept study led us to consider that the AAbs as well as the immune monitoring of specific peripheral lymphocyte subsets should be investigated as potential biomarkers of autoimmunity and (potentially) treatment response in patients with NSCLC receiving PD-1/PD-L1 immune-checkpoint blockade. Furthermore, to identify a possible mechanistic scenario, correlative biological/clinical studies including inflammatory and angiogenesis studies coupled with deep immunological characterization should be performed to characterize the effects produced by PD-1/PD-L1 immune-checkpoint blockade.

## Data availability statement

The raw data supporting the conclusions of this article will be made available by the authors, without undue reservation.

## Ethics statement

Ethical review and approval was not required for the study on human participants in accordance with the local legislation and institutional requirements. The patients/participants provided their written informed consent to participate in this study.

## Author contributions

PC and AFam conceived the work. GB, CG, BO, AFal, CR, and NC collected and reviewed the data. VN, MS, and CB performed the whole analysis and gave conceptual input to the work. AFam, DA, RG, MM, RS, and PC performed most of the clinical and laboratory activity on patients and patient samples. LP, AG, PTas, PTag, MC, AFal and LM deeply reviewed the work and gave clinical and scientific input for research development. AFam, MS, CB, and PC prepared the figures and manuscript draft, which has been revised and approved by all authors in its final form. All authors contributed to the article and approved the submitted version.

## Funding

M Azgomi Shekarkar was supported by grants from the Italian Association for Cancer Research (AIRC) within the My First AIRC Grant 2020 (n. 24534, 2021/2025) PI: CB.

## Conflict of interest

The authors declare that the research was conducted in the absence of any commercial or financial relationships that could be construed as a potential conflict of interest.

## Publisher’s note

All claims expressed in this article are solely those of the authors and do not necessarily represent those of their affiliated organizations, or those of the publisher, the editors and the reviewers. Any product that may be evaluated in this article, or claim that may be made by its manufacturer, is not guaranteed or endorsed by the publisher.
