# Supplementary material for: PD-1/PD-L1 immune-checkpoint blockade induces immune effector cell modulation in metastatic non-small cell lung cancer patients: A single-cell flow cytometry approach
Source: Front Oncol. 2022 Sep 14;12:911579. doi: 10.3389/fonc.2022.911579 (PMC9515511; doi:10.3389/fonc.2022.911579)
Supplement: Supplementary file 1 [file DataSheet_1.docx]

Supplementary Material

## Supplementary Figures


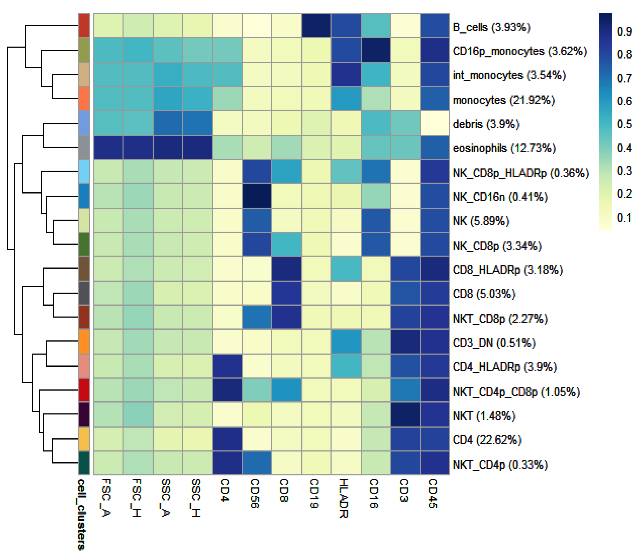

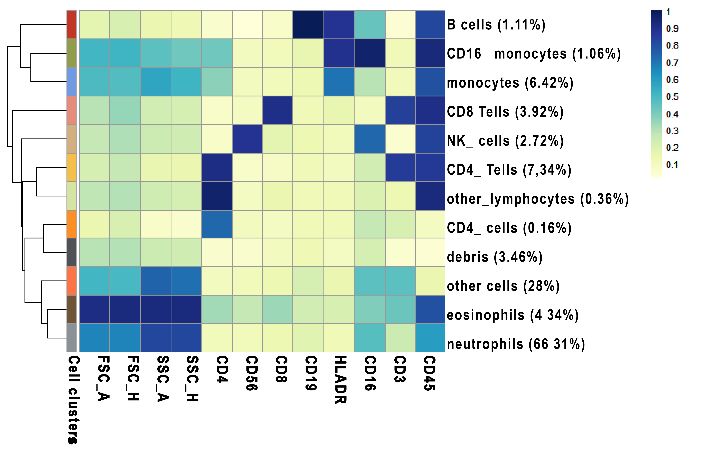
**
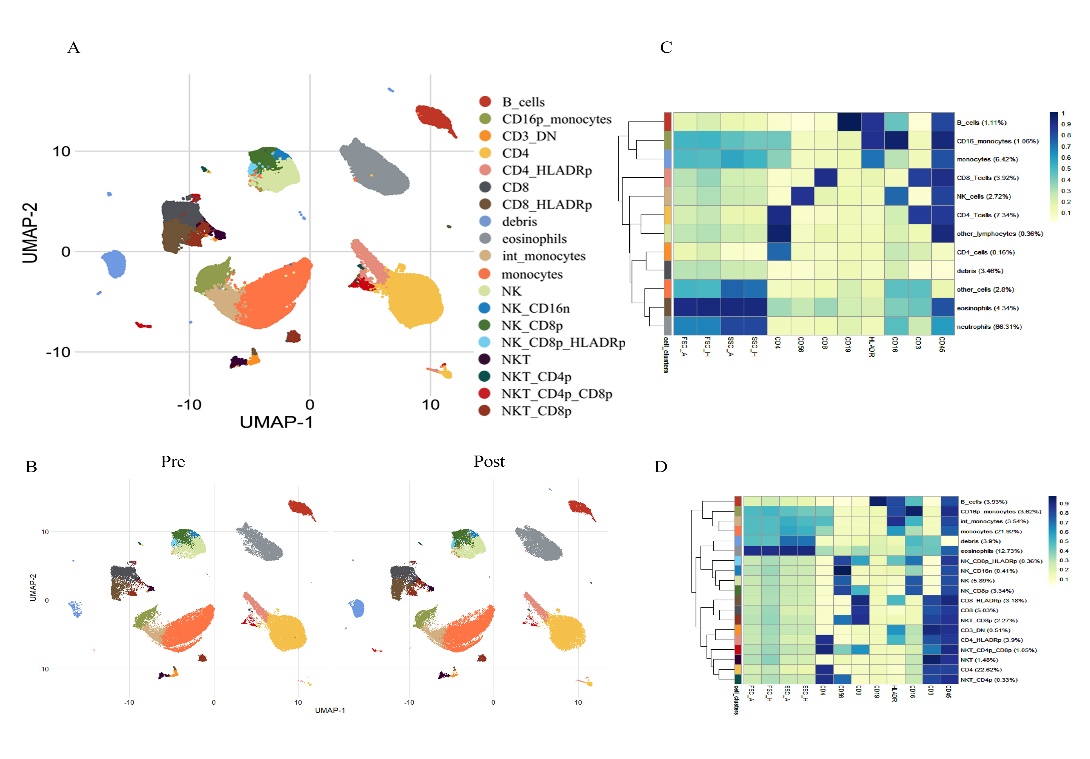
**

**Supplementary Figure 1.** clustering and cell subset Identification: A: after normalization and dimensionally reduction, all the cells have been clustered into 19 sub-clusters. An initial clustering step to remove neutrophils has been used. B The final clustering of different subsets could clearly show that no batch effect related to treatment could be identified (no new clusters appeared after treatment). C: based on cell marker, the initial cell subset clustering using FlowSOM is performed on all the cells, D heatmap showing markers expression on the final clusters.


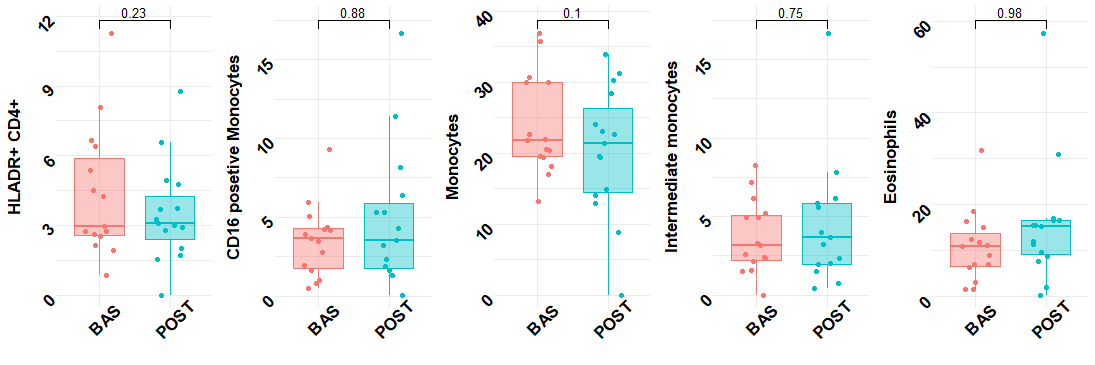

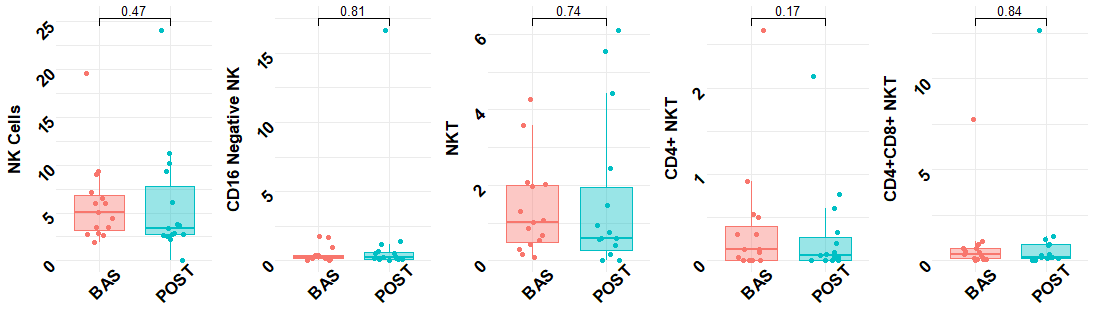

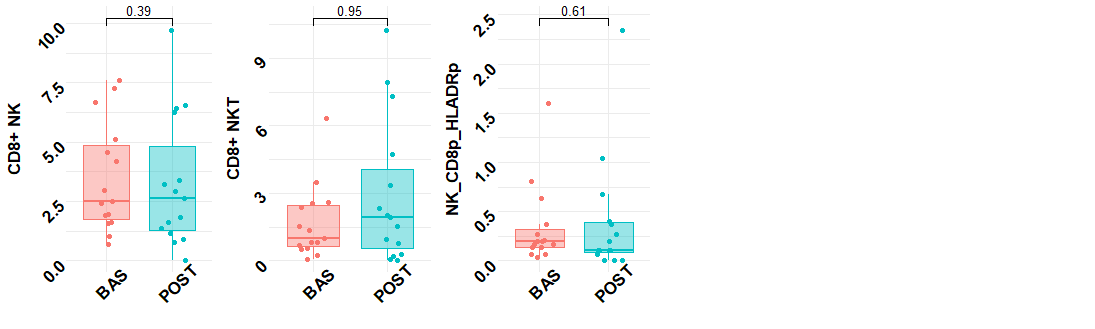


Condition

BAS

POST

**Supplementary Figure 2.** PBMCs clusters identified by FlowCT evaluated according to treatment time (baseline vs. post three treatment courses)

**Supplementary Figure 3.** **peripheral blood cell count from OM-RC and RT-SI centers.**

A: CBC in baseline and after 3 treatment courses in both discovery and validation datasets. B: abundance in CD4 T cells in Peripheral blood of patients from RT-SI center.

**Supplementary Figure 4: subset change and survival correlation:** simple linear correlation between all 19 cell subsets and survival OS (months): X-axes report the abundance of the different cell subsets, and Y-axes report OS (in months) statistical results are shown in supplementary table 1.

|  | B_cells | | CD16p_monocytes | | | CD3_DN | | | CD4 | | | CD4_HLADRp | | | CD8 | | | CD8_HLADRp | | | Eosinophils | | | int_monocytes | | | Monocytes | | |
| --- | --- | --- | --- | --- | --- | --- | --- | --- | --- | --- | --- | --- | --- | --- | --- | --- | --- | --- | --- | --- | --- | --- | --- | --- | --- | --- | --- | --- | --- |
|  | Before | After | Before | After | Before | | After | Before | | After | Before | | After | Before | | After | Before | | After | Before | | After | Before | | After | Before | | After |  |
| R squared | 0.01 | 0.02 | 0.59 | 0.20 | 0.56 | | 0.09 | 0.03 | | 0.16 | 0.12 | | 0.27 | 0.17 | | 0.22 | 0.55 | | 0.07 | 0.08 | | 0.06 | 0.00 | | 0.04 | 0.19 | | 0.07 |  |
| P value | 0.76 | 0.71 | 0.01 | 0.17 | 0.01 | | 0.36 | 0.63 | | 0.22 | 0.30 | | 0.10 | 0.21 | | 0.15 | 0.01 | | 0.44 | 0.39 | | 0.48 | 0.88 | | 0.57 | 0.18 | | 0.43 |  |
|  | NK | | NK_CD16n | | | NK_CD8p | | | NK_CD8p_HLADRp | | | NKT | | | NKT_CD4p | | | NKT_CD4p_CD8p | | | NKT_CD8p | | | Neutrophils | | | | | |
|  | Before | After | Before | After | Before | | After | Before | | After | Before | | After | Before | | After | Before | | After | Before | | After | Before | | After |  |  |  |  |
| R squared | 0.03 | 0.07 | 0.00 | 0.00 | 0.04 | | 0.14 | 0.13 | | 0.05 | 0.10 | | 0.38 | 0.04 | | 0.01 | 0.00 | | 0.01 | 0.01 | | 0.01 | 0.02 | | 0.01 |  |  |  |  |
| P value | 0.64 | 0.44 | 0.96 | 0.85 | 0.58 | | 0.25 | 0.28 | | 0.51 | 0.35 | | 0.04 | 0.53 | | 0.77 | 0.85 | | 0.82 | 0.74 | | 0.82 | 0.65 | | 0.82 |  |  |  |  |

**Supplementary Table 1: the statistical result of the correlation between subset change and survival:** r squared and p-value of all subsets tested for simple linear correlation, p value< 0.01 considered as a significant correlation

**Supplementary Figure 5.** **Survival analysis of changing subset frequency Fold change during therapy**. Statistical analysis has been performed by Log-rank (Mantel-Cox) test, and p-value and chi-square are shown in each plot. P-value <0.01 consider as statistically significant [GP: 0.12(ns),0.03(*),<0.002(**)]

**Supplementary Figure 6: 19 different subset change** **in baseline and after three treatment curse in 2 groups of study ( serum antinuclear-antibodies positive or negative):**

% Of subset frequency in two different groups based on positive or negative Aab in baseline and after three treatment curs was shown. In order to find a significant statistical difference between baseline and after three treatment was calculated based on Dunn’s multiple comparisons with correction. P-value <0.05 consider a statistically significant change. The X-axes are found in the % of cell subsets in different study groups.

**Supplementary Figure 7.** Changes in cell subset frequency according to the appearance of irAEs. Significant changes are indicated with * based on p value< 0.1 (due to low numbers we decided to be more permissive). Statistical analysis has been performed using the Mann-Whitney test.

**Supplementary Figure 8.** Kaplan Meier curves report survival results according to HLA alleles. Comparisons have been done according to Log-rank (Mantel-Cox) test.

**Supplementary Figure 9.** A: Correlation analysis of cell subset changes during therapy based on different HLA types. None of the comparisons reached a statistical significance.
